# Supplementary material for: Mixed Methods Studies on Breastfeeding: A Scoping Review
Source: Healthcare (Basel). 2025 Mar 27;13(7):746. doi: 10.3390/healthcare13070746 (PMC11988830; doi:10.3390/healthcare13070746)
Supplement: Supplementary file 1 [file healthcare-13-00746-s001.zip › Additional 1_Table S1_Terms for the strategic search in information sources.pdf]

Table S1 – Terms for the strategic search in information sources.

Database	Search Strategy
Medline<br>(Via Pubmed)	(Women [mesh terms] OR Girls [tiab] OR Girl [tiab] OR Woman [tiab] OR Women's Groups [tiab] OR Women Groups[tiab] OR Women's Group [tiab] OR Pregnant Women [mesh terms])<br># **2** (Breast Feeding [mesh terms] OR Breastfed [tiab] OR Breastfeeding [tiab] OR Breast Fed [tiab] OR Breast Feeding, Exclusive [tiab] OR Exclusive Breast Feeding [tiab] OR Breastfeeding, Exclusive [tiab] OR Exclusive Breastfeeding) **AND** (Maternal-Child Health Centers [mesh terms] OR Center, Maternal-Child Health [tiab] OR Centers, Maternal-Child Health [tiab] OR Health Center, Maternal-Child [tiab] OR Health Centers, Maternal-Child [tiab] OR Maternal Child Health Centers [tiab] OR Maternal-Child Health Center [tiab] OR Community Health Centers [mesh terms] OR Center, Community Health [tiab] OR Centers, Community Health [tiab] OR Community Health Center [tiab] OR Health Center, Community [tiab] OR Health Centers, Community OR Hospitals, Maternity [mesh terms] OR Maternity Hospitals [tiab] OR Hospital, Maternity [tiab] OR Maternity Hospital [tiab]) **AND** (Mixed methods)
EMBASE	('female' OR 'females' OR 'woman' OR 'women' OR 'female') **AND** ('breast feeding' OR 'feeding, breast' OR 'breast feeding') **AND** ('health center' OR 'center, health' OR 'clinic, health' OR 'community health center' OR 'community health centers' OR 'health care center' OR 'health care centre' OR 'health centre' OR 'health clinic' OR 'health institute' OR 'health resort' OR 'health resorts' OR 'health service center' OR 'health unit' OR 'healthcare center' OR 'healthcare centre' OR 'resort, health' OR 'health center') **AND** (Mixed methods)
Cochrane	('Women' OR 'Girls' OR 'Girl' OR 'Woman' OR 'Women's Groups' OR 'Women Groups' OR 'Women's Group' OR 'Pregnant Women') **AND** ('Breast Feeding' OR 'Breastfed' OR 'Breastfeeding' OR 'Breast Fed' OR 'Breast Feeding, Exclusive' OR 'Exclusive Breast Feeding' OR 'Breastfeeding, Exclusive' OR 'Exclusive Breastfeeding') **AND** ('Maternal-Child Health Centers' OR 'Center, Maternal-Child Health' OR 'Centers, Maternal-Child Health' OR 'Health Center, Maternal-Child' OR 'Health Centers, Maternal-Child' OR 'Maternal Child Health Centers' OR 'Maternal-Child Health Center' OR 'Community Health Centers' OR 'Center, Community Health' OR 'Centers, Community Health' OR 'Community Health Center' OR 'Health Center, Community' OR 'Health Centers, Community' OR 'Hospitals, Maternity' OR 'Maternity Hospitals' OR 'Hospital, Maternity' OR 'Maternity Hospital') **AND** (Mixed methods)
CINHAL<br>(via EBSCOhost)	(MH '**Woman**' OR 'Woman'OR MH '**Women'**OR 'Girls' OR 'Girl' OR 'Women's Groups' OR 'Women Groups' OR 'Women's Group' OR 'Pregnant Women') **AND** (MH **'breast-feeding'** OR 'Breast Feeding' OR 'Breastfed' OR 'Breastfeeding' OR 'Breast Fed' OR 'Breast Feeding, Exclusive' OR 'Exclusive Breast Feeding' OR 'Breastfeeding, Exclusive' OR 'Exclusive Breastfeeding') **AND (**MH **'motherhood'** OR 'Maternal-Child

	Health Centers' OR 'Center, Maternal-Child Health' OR 'Centers, Maternal-Child Health' OR 'Health Center, Maternal-Child' OR 'Health Centers, Maternal-Child' OR 'Maternal Child Health Centers' OR 'Maternal-Child Health Center' OR 'Community Health Centers' OR 'Center, Community Health' OR 'Centers, Community Health' OR 'Community Health Center' OR 'Health Center, Community' OR 'Health Centers, Community' OR 'Hospitals, Maternity' OR 'Maternity Hospitals' OR 'Hospital, Maternity' OR 'Maternity Hospital') **AND** (Mixed methods)
Web of Science	('Women'OR 'Girls' OR 'Girl' OR 'Woman' OR 'Women's Groups' OR 'Women Groups' OR 'Women's Group' OR 'Pregnant Women') **AND** ('Breast Feeding' OR 'Breastfed' OR 'Breastfeeding' OR 'Breast Fed' OR 'Breast Feeding, Exclusive' OR 'Exclusive Breast Feeding' OR 'Breastfeeding, Exclusive' OR 'Exclusive Breastfeeding') **AND** ('Maternal-Child Health Centers' OR 'Center, Maternal-Child Health' OR 'Centers, Maternal-Child Health' OR 'Health Center, Maternal-Child' OR 'Health Centers, Maternal-Child' OR 'Maternal Child Health Centers' OR 'Maternal-Child Health Center' OR 'Community Health Centers' OR 'Center, Community Health' OR 'Centers, Community Health' OR 'Community Health Center' OR 'Health Center, Community' OR 'Health Centers, Community' OR 'Hospitals, Maternity' OR 'Maternity Hospitals' OR 'Hospital, Maternity' OR 'Maternity Hospital') **AND** (Mixed methods)
BVS	('Women'OR 'Girls' OR 'Girl' OR 'Woman' OR 'Women's Groups' OR 'Women Groups' OR 'Women's Group' OR 'Pregnant Women') **AND** ('Breast Feeding' OR 'Breastfed' OR 'Breastfeeding' OR 'Breast Fed' OR 'Breast Feeding, Exclusive' OR 'Exclusive Breast Feeding' OR 'Breastfeeding, Exclusive' OR 'Exclusive Breastfeeding') **AND** ('Maternal-Child Health Centers' OR 'Center, Maternal-Child Health' OR 'Centers, Maternal-Child Health' OR 'Health Center, Maternal-Child' OR 'Health Centers, Maternal-Child' OR 'Maternal Child Health Centers' OR 'Maternal-Child Health Center' OR 'Community Health Centers' OR 'Center, Community Health' OR 'Centers, Community Health' OR 'Community Health Center' OR 'Health Center, Community' OR 'Health Centers, Community' OR 'Hospitals, Maternity' OR 'Maternity Hospitals' OR 'Hospital, Maternity' OR 'Maternity Hospital') **AND** (Mixed methods)
SCOPUS	('Women'OR 'Girls' OR 'Girl' OR 'Woman' OR 'Women's Groups' OR 'Women Groups' OR 'Women's Group' OR 'Pregnant Women') **AND** ('Breast Feeding' OR 'Breastfed' OR 'Breastfeeding' OR 'Breast Fed' OR 'Breast Feeding, Exclusive' OR 'Exclusive Breast Feeding' OR 'Breastfeeding, Exclusive' OR 'Exclusive Breastfeeding') **AND** (Mixed methods)
BDTD	(Women OR Girls OR Girl OR Woman OR Women's Groups OR Women Groups OR Women's Group OR Pregnant Women **AND** Breast Feeding OR Breastfed OR Breastfeeding OR Breast Fed OR Breast Feeding, Exclusive OR Exclusive Breast Feeding OR Breastfeeding, Exclusive OR Exclusive Breastfeeding **AND** Mixed methods)
