# Supplementary material for: Mixed Methods Studies on Breastfeeding: A Scoping Review
Source: Healthcare (Basel). 2025 Mar 27;13(7):746. doi: 10.3390/healthcare13070746 (PMC11988830; doi:10.3390/healthcare13070746)
Supplement: Supplementary file 1 [file healthcare-13-00746-s001.zip › Additional 2 - Chart S1 - Evaluation of studies using the Mixed Methods Appraisal Tool - MMAT.pdf]

| | | GERAL | | ESPECÍFICOS | | | | |
|---|---|---|---|---|---|---|---|---|
| Nº | Autor/Ano | Questão 1* | Questão 2** | Questão 3*** | Questão 4**** | Questão 5***** | Questão 6****** | Questão 7******* |
| 1 | Tucker et al 2011 | ✓ | ✓ | ✓ | ✗ | ✗ | ✓ | ✓ |
| 2 | White et al, 2012 | ✓ | ✓ | ✗ | ✗ | ✗ | ✓ | ✗ |
| 3 | Agunbiade, 2012 | ✓ | ✓ | ✓ | ✗ | ✗ | ✓ | ✓ |
| 4 | Ingram, 2013 | ✓ | ✓ | ✓ | ✗ | ✗ | ✓ | ✗ |
| 5 | Dörnemann, 2013 | ✓ | ✓ | ✓ | ✗ | ✗ | ✓ | ✓ |
| 6 | Kadakia, 2015 | ✓ | ✓ | ✗ | ✗ | ✗ | ✓ | ✓ |
| 7 | Maonga, 2016 | ✓ | ✓ | ✗ | ✗ | ✗ | ✓ | ✓ |
| 8 | Murray, 2016 | ✓ | ✓ | ✗ | ✗ | ✗ | ✓ | ✓ |
| 9 | Kamoun, 2018 | ✓ | ✓ | ✓ | ✗ | ✗ | ✓ | ✓ |
| 10 | Feenstra, 2018 | ✓ | ✓ | ✗ | ✗ | ✗ | ✓ | ✓ |
| 11 | Chen, 2019 | ✓ | ✓ | ✗ | ✗ | ✗ | ✓ | ✓ |
| 12, | Ramos, 2019 | ✓ | ✓ | ✗ | ✗ | ✗ | ✓ | ✗ |
| 13 | Hall, 2015 | ✓ | ✓ | ✗ | ✗ | ✗ | ✓ | ✓ |
| 14 | Kohan, 2019 | ✓ | ✓ | ✓ | ✗ | ✗ | ✗ | ✓ |
| 15 | Zhou, 2020 | ✓ | ✓ | ✓ | ✗ | ✗ | ✓ | ✓ |
| 16 | Juntereal, 2020 | ✓ | ✓ | ✗ | ✗ | ✗ | ✓ | ✗ |
| 17 | Abraham, 2020a | ✓ | ✓ | ✗ | ✗ | ✗ | ✓ | ✗ |
| 18 | Abraham, 2020b | ✓ | ✓ | ✗ | ✗ | ✗ | ✓ | ✗ |
| 19 | Shobo, 2020 | ✓ | ✓ | ✓ | ✗ | ✗ | ✓ | ✓ |
| 20 | Ericson, 2020 | ✓ | ✓ | ✓ | ✗ | ✗ | ✓ | ✓ |
| 21 | Witten, 2020 | ✓ | ✓ | ✓ | ✓ | ✓ | ✓ | ✓ |
| 22 | Hernández-Cordero, 2022 | ✓ | ✓ | ✗ | ✗ | ✗ | ✓ | ✓ |
| 23 | Kubuga, 2023 | ✓ | ✓ | ✓ | ✗ | ✗ | ✓ | ✓ |
| 24 | Hookway, 2023 | ✓ | ✓ | ✗ | ✗ | ✗ | ✓ | ✗ |

| | | | | | | | | |
|---|---|---|---|---|---|---|---|---|
| 25 | Kwan, 2022 | ✓ | ✓ | ✓ | ✗ | ✗ | ✓ | ✓ |
| 26 | Irmawati, 2022 | ✓ | ✓ | ✗ | ✗ | ✗ | ✓ | ✓ |
| 27 | Nuampa, 2022 | ✓ | ✓ | ✗ | ✗ | ✗ | ✓ | ✓ |
| 28 | Jonsdottir, 2022 | ✓ | ✓ | ✗ | ✗ | ✗ | ✓ | ✓ |
| 29 | Jiravisitkul, 2022 | ✓ | ✓ | ✗ | ✗ | ✗ | ✓ | ✓ |
| 30 | Felder, 2022 | ✓ | ✓ | ✓ | ✓ | ✓ | ✓ | ✓ |
| 31 | Saade, 2022 | ✓ | ✓ | ✗ | ✗ | ✗ | ✓ | ✓ |
| 32 | Van Breevoort, 2021 | ✓ | ✓ | ✗ | ✗ | ✗ | ✓ | ✓ |
| 33 | Gordon, 2021 | ✓ | ✓ | ✗ | ✗ | ✗ | ✓ | ✓ |
| 34 | Nkrumah, 2021 | ✓ | ✓ | ✗ | ✗ | ✗ | ✓ | ✓ |
| 35 | Papautsky, 2021 | ✓ | ✓ | ✗ | ✗ | ✗ | ✓ | ✗ |
| 36 | Kim, 2017 | ✓ | ✓ | ✗ | ✗ | ✗ | ✓ | ✓ |

O sinal ✓ (em verde) é "SIM" e o sinal ✗ (vermelho) é "NÃO".
*Existem questões de pesquisa claras?
** Os dados recolhidos permitem responder às questões de investigação?
*** Existe uma justificativa adequada para usar um desenho de métodos mistos para abordar a questão da pesquisa?
**** Os diferentes componentes do estudo estão efetivamente integrados para responder à questão de pesquisa?
***** Os resultados da integração dos componentes qualitativos e quantitativos são interpretados de forma adequada?
****** As divergências e inconsistências entre resultados quantitativos e qualitativos são abordadas de forma adequada?
******* Os diferentes componentes do estudo atende aos critérios de qualidade de cada tradição dos métodos envolvidos?
